# Exosomes from human umbilical cord mesenchymal stem cells attenuate the inflammation of severe steroid-resistant asthma by reshaping macrophage polarization

**DOI:** 10.1186/s13287-021-02244-6

**Published:** 2021-03-24

**Authors:** Bing Dong, Chao Wang, Jing Zhang, Jinrong Zhang, Yinuo Gu, Xiaoping Guo, Xu Zuo, He Pan, Alan Chen-Yu Hsu, Guoqiang Wang, Fang Wang

**Affiliations:** 1 Department of Pathogeny Biology, College of Basic Medical Sciences, Jilin University, Changchun, 130021 China; 2 Priority Research Centre for Asthma and Respiratory Diseases, Hunter Medical Research Institute and the University of Newcastle, Newcastle, NSW 2305 Australia

**Keywords:** Mesenchymal stem cell, Exosome, Macrophage polarization, Inflammation, Severe steroid-resistant asthma

## Abstract

**Background:**

Severe, steroid-resistant asthma (SSRA) is a serious clinical problem in asthma management. Affected patients have severe clinical symptoms, worsened quality of life, and do not respond to steroid, a mainstay steroid treatment of asthma. Thus, effective therapies are urgently needed. Exosomes derived from mesenchymal stem cell (MSC-Exo) has become attractive candidates for the lung inflammatory diseases through its immunomodulatory effects. In this study, we explored the therapeutic effects of MSC-Exo in SSRA and identified the therapeutic mechanism of MSC-Exo.

**Method:**

Exosomes from human umbilical cord mesenchymal stem cell (hUCMSC) were isolated and characterized by transmission electron microscopy, nanoparticle tracking analysis and flow cytometry analysis. Effects of MSC-Exo on airway hyper responsiveness (AHR), inflammation, histopathology, and macrophage polarization in SSRA in mice were evaluated. Systematic depletion of macrophages determined the role of macrophages in the therapeutic effect of SSRA in mice. LPS-stimulated RAW 264.7 cell model was constructed to determine the underlying mechanism of MSC-Exo on macrophage polarization. qRT-PCR, Western blotting, immunofluorescence, and flow cytometry were performed to evaluate the expression of M1 or M2 markers. Tandem mass tags (TMT)-labeled quantitative proteomics were applied to explore the central protein during the regulation effect of MSC-Exo on macrophage polarization. Knockdown and overexpression of TRAF1 were used to further clarify the role of the central protein on macrophage polarization.

**Result:**

We successfully isolated and characterized exosomes from hUCMSCs. We verified that the intratracheal administration of MSC-Exo reversed AHR, histopathology changes, and inflammation in SSRA mice. Systematic depletion of macrophages weakened the therapeutic effect of MSC-Exo. We found that MSC-Exo treatment inhibited M1 polarization and promoted M2 polarization in LPS-stimulated RAW 264.7 cells. Subsequently, tumor necrosis factor receptor-associated factor 1 (TRAF1) was determined as the central protein which may be closely related to the regulation of macrophage polarization from TMT-labeled quantitative proteomics analysis. Knockdown and overexpression of TRAF1 demonstrated that the effect of MSC-Exo treatment on macrophage polarization, NF-κB and PI3K/AKT signaling was dependent on TRAF1.

**Conclusion:**

MSC-Exo can ameliorate SSRA by moderating inflammation, which is achieved by reshaping macrophage polarization via inhibition of TRAF1.

## Background

Asthma is a heterogeneous disease with variable clinical and pathological characteristics, suggesting that asthma has multiple phenotypes. Severe, steroid-resistant asthma (SSRA) has different pathological features to conventional allergic asthma and is characterized by frequent acute exacerbations by pathogens, severe symptoms, lower lung function, and greater tissue inflammation [1]. Patients with SSRA subtype do not respond to mainstay treatment even with high doses of steroids and suffer a worse quality of life [2]. Immune cells and the disease-associated cytokines they secret are important in the pathogenesis of SSRA. These mediators such as tumor necrosis factor alpha (TNF-α) and interleukin-6 (IL-6) exacerbate airway inflammation and lung tissue damage [3–5]. Macrophages are the most abundant immune cells in lung tissue (approximately 70% of all immune cells) and play central roles in regulating airway inflammation and airway remodeling [6, 7]. Macrophages are categorized into classic activated (M1) and alternative activated (M2) phenotypes based on the expression of specific cytokines and surface markers [8]. During the acute exacerbation of SSRA, M1 macrophages have been shown to secrete large amounts of inflammatory mediators (include TNF-α, IL-1β, IL-6, inducible nitric oxide synthase (iNOS)) [9]. These inflammatory factors are associated with neutrophil-rich infiltration, airway hyper-responsiveness (AHR) and airway remodeling [2]. M2 macrophages (M2 biomarkers include arginase-1 (Arg-1), CD206, and IL-10) produce an anti-inflammatory environment that also promotes tissue repair [10]. Hence, M1 and M2 phenotypic switch might be a potentially novel therapeutic avenue for the treatment of SSRA.

Mesenchymal stem cells (MSCs) hold great promise as a feasible and effective biological treatment for inflammation-related diseases [11, 12]. MSCs have been shown to elicit immunomodulatory and tissue repair effects in lung inflammatory diseases, including reducing airway inflammation and ameliorating lung function [13–15]. Several studies have demonstrated that macrophage-derived pro-inflammatory cytokines were reduced by MSCs through modulating macrophage phenotype in vivo and vitro [16]. However, the underlying mechanism remains unclear. Despite the physiological condition of the recipient, tissue has been greatly improved after MSCs therapy, accumulating evidence showed that MSCs after systemic transplantation were quickly trapped in the pulmonary vascular bed due to their large size and failed to reach the target sites. The therapeutic effect by MSCs in the absence of significant engraftment at the target sites suggesting that the mechanism of MSCs therapeutic function is mainly paracrine factors [17, 18]. Exosomes are an important component of the secretory pathway of MSCs and carry important intracellular factors that regulate immune responses and cell biological functions [19, 20]. Moreover, compared with MSCs-based therapy, MSCs-exosomes (MSC-Exo)-based cell-free therapy has the advantages of non-tumorigenicity, high stability, and no vascular obstruction [21, 22]. Several recent studies have shown that MSC-Exo can repair tissue damage and prevent autoimmune diseases [16, 17]. However, it remains unknown if MSC-Exo could govern macrophages to create an anti-inflammatory environment in SSRA.

In this study, we demonstrated the immunomodulatory effect of human umbilical cord MSC-Exo in a severe, steroid-resistant asthma murine model. We investigated the effects of MSC-Exo on macrophage polarization and inflammation both in SSRA mice and LPS-stimulated cell model. Furthermore, tandem mass tags (TMT)-labeled quantitative proteomics was performed to explore and characterize the underlying biological mechanism of the therapeutic effect of MSC-Exo. This study presents a potentially new therapeutic strategy for severe, steroid-resistant asthma.

## Materials and methods

### Isolation and identification of hUCMSC-derived exosomes

Human umbilical cord-derived mesenchymal stem cells (hUCMSCs) were purchased from ScienCell (San Diego, USA, Cat No. 7530) and cultured in Mesenchymal Stem Cell Medium (ScienCell, San Diego, USA, Cat No. 7501) at 5% CO_2_ and 37 °C. The cells at passage four were used for experiments. Exosomes were collected from the supernatants of hUCMSCs using exoEasy Maxi Kit (Qiagen, Germany) according to product protocols, and ultrastructure and shape were confirmed by transmission electron microscopy (Tecnai Spirit TEM T12, FEI). Protein concentration of exosomes was determined by BCA assay. The size distribution and nanoparticle concentration of exosomes were detected using nanoparticle tracking analysis (NTA, ZetaView PMX 120). Surface markers CD63 (557,288, FITC, BD, San Jose, USA), CD81 (555,676, PE, BD, San Jose, USA) of exosomes were detected by flow cytometry (Accuri C6, BD, San Jose, USA).

### Establishment of an OVA/CFA-induced severe, steroid-resistant asthma (SSRA) murine model and treatment

All the animal experimental procedures were performed in accordance with the guidelines (protocol No. 2015-34) and approved by Animal Care and Use Committee of Jilin University. BALB/c female mice (18–22 g) were purchased from HFK Bioscience (Beijing, China). A murine model of SSRA was constructed using ovalbumin/complete Freund’s adjuvant (OVA/CFA, Sigma-Aldrich, USA) according to an optimized protocol based on previous studies [23, 24]. Mice were divided into four groups: (1) control group (*n* = 12), (2) OVA/CFA group (*n* = 12), (3) dexamethasone (DEX) group (*n* = 12), and (4) MSC-Exo group (*n* = 12). Mice were sensitized on day 0, 7, and 14 with intraperitoneal injection of OVA/CFA (20 μg OVA in 75 μl CFA and 25 μl PBS). The mice were challenged with 2% OVA via using DSI Buxco Inhalation Exposure system (DSI Buxco, USA) at day 21, 22, and 23. Intratracheal administration of exosomes (100 μg in 50 μl PBS per mouse) was carried out at day 21. Mice from control group were sensitized, challenged, and administrated with same volume of PBS at the same point in time. Mice from DEX group were treated with dexamethasone (2 mg/kg, Sigma-Aldrich, USA) by intraperitoneal injection at day 21, 22, and 23. All mice were anesthetized and euthanized for the following procedures at day 24.

The macrophage depletion experiment was modified according to the methods in previous studies [16, 25]. To deplete macrophages, mice were administrated with Clodronate Liposomes (C-lipo, 0.2 ml per mouse) or PBS-liposomes (P-lipo, 0.2 ml per mouse) (LIPOSOMA, Amsterdam, The Netherlands, CP-005-005) intraperitoneally on day 20, 21, and 22. Mice were divided into four groups: (1) OVA/CFA+P-lipo group (*n* = 6), (2) OVA/CFA+P-lipo+Exo group (*n* = 6), (3) OVA/CFA+C-lipo group (*n* = 6), and (4) OVA/CFA+C-lipo+Exo group (*n* = 6). All mice were anesthetized and euthanized for the following procedures at day 24.

### Measurement of airway hyper-responsiveness (AHR)

Mice were anesthetized with pentobarbital (40 mg/kg) by intraperitoneal injection. When the mice entered a deeply anesthetized state, they were intubated by airway for ventilation (tidal volume of 8 ml/kg at a rate of 140 breaths/min) and quickly connected to the DSI Buxco FinePoint RC system (DSI Buxco, USA) [26]. Subsequently, they were exposed to increasing doses of acetyl-β-methylcholine chloride (Sigma-Aldrich, USA) at 6.25, 12.5, 25, and 50 mg/ml or PBS for 2 min. Respiratory system resistance and dynamic compliance were obtained from the software of DSI Buxco system. The results are exhibited as an average value of maximal resistance after each dose of methacholine minus baseline (PBS alone) resistance.

### Histopathology evaluation

For histopathological evaluation, lung tissue was collected, fixed with paraformaldehyde and embedded with paraffin. Subsequently, 5-μm thick sections were cut and stained with hematoxylin and eosin (HE). Evaluation of peribronchial inflammation level (0–4) of lung tissue was performed as previously described [27]. The histological score of all samples were determined by two assessors who were blind to the experimental groups.

### LPS stimulation and MSC-Exo treatment in RAW 264.7 cells

RAW 264.7 cells and the corresponding complete medium were purchased from Zhong Qiao Xin Zhou Biotechnology Co., Ltd. (Shanghai, China). Cells were cultured at 5% CO_2_ and 37 °C in a humidified cell incubator machine. The experiment was divided into five groups: the PBS group (control), cells treated with PBS; the LPS group (LPS), cells treated with LPS (1 μg/ml) for 12 h; the LPS+MSC-Exo groups, cells treated with exosomes (10, 20, 40 μg/ml) for 24 h after the treatment of LPS.

### Sample preparation and analysis of tandem mass tags (TMT)-labeled quantitative proteomics

RAW 264.7 cells from LPS group and LPS+MSC-Exo group were collected for the TMT-labeled quantitative proteomics analysis. After the quality control of the protein samples, the lyophilized samples were labeled with TMT. The proteomics analysis was performed using a Q-Exactive mass spectrometer (Thermo, USA) by oebiotech (Shanghai, China). The obtained LC-MS/MS data were processed using Proteome Discover 2.4 (Thermo, USA) and the credible proteins were screened according to the criteria of Score Sequest HT > 0 and unique peptide ≥ 1, and the blank value removed. Based on the credible protein, Student’s *t* test was performed to identify the significant difference of proteins in two groups. Fold change more than 1.5 or less than 0.67 and *p* value less than 0.05 were considered to be significantly different. The volcano plot of differentially expressed proteins was performed using R software (version 4.2) ggplot2 package (version 3.2.2). Annotation information of each identified protein was extracted using Uniprot database. The cluster analysis of differential protein expression levels was conducted using R software (version 4.2) pheatmap package (version 1.0.12). Pathway enrichment analysis of differential proteins was performed based on the KEGG database using R software (version 4.2) ggplot2 package (version 3.2.2).

### Enzyme-linked immunosorbent assay (ELISA)

The levels of TNF-α, IL-6, IL-1β, and IL-10 in supernatants from lung tissue homogenate of mice and supernatants from RAW 264.7 cells were measured using ELISA kits (Lianke, Hangzhou, China). The assay was conducted following the guide of manufacturer’s protocols. The absorbance was detected using Epoch microplate reader (BioTek, USA) at 450 nm. The reference length was 570 nm.

### Western blotting

Protein samples from lung tissues and RAW 264.7 cells were extracted using Minute Total Protein Extraction Kit (Invent Biotechnologies, USA). The content of each sample was determined by BCA protein assay kit (Beyotime, Shanghai, China). Denatured protein samples (20 μg) were separated by 10% SDS-PAGE (Yeasen Biotech, Shanghai, China) and trans-printed onto PVDF membranes. The membranes were then blocked with 5% non-fat milk for 1 h and subsequently reacted with the specific primary antibodies (1:1000): iNOS, Arg1, p65, p-p65 (Abcam, Cambridge, UK), TRAF1, PI3K (Cell Signaling Technology, Beverly, USA), AKT, p-AKT, p-PI3K, and β-actin (Biossen, Beijing, China) overnight at 4 °C. The membranes were washed for six times in Tris Buffered saline Tween (TBST), 5 min each, and were incubated with goat derived anti-rabbit (1:20000) or anti-rat secondary antibody (1:2000) for 1 h under room temperature. After the membranes were washed with TBST for 6 times, 5 min each, the bands were developed using SuperSignal Chemiluminescent Substrate kit (Thermo Scientific, Rockford, USA) and detected by GeneGnome XRQ (Gene Company, Shanghai, China). The bands were analyzed using ImageJ software.

### Quantitative real-time PCR (qRT-PCR)

Total RNA from lung tissues and RAW 264.7 cells were extracted using AxyPrep Multisource Total RNA kit (AXYGEN, USA). RNA was transcribed to cDNA using first strand cDNA synthesis kit (Novoprotein, Shanghai, China). The gene transcripts were detected using Novostart SYBER qPCR SuperMix Plus (Novoprotein, Shanghai, China) with a 7300 plus real-time PCR system (Applied Biosystems, USA) according to the manufacturer’s protocols. GAPDH was used as the internal reference. The sequences of primers were used as follows: mouse GAPDH forward: 5′- AGGTCGGTGTGAACGGATTTG-3′, reverse: 5′-CGCCACGAGCAGGAATGAGAAG-3′; mouse TNF-α forward: 5′-GCATGATCCGAGATGTGGAACTGG-3′, reverse: 5′-CGCCACGAGCAGGAATGAGAAG-3′; mouse IL-6 forward: 5′-AATTTCCTCTGGTCTTCTGGAGT-3′, reverse: 5′- GTGACTCCAGCTTATCTCTTGGT-3′; mouse IL-1β forward: 5′-GCAGAGCACAAGCCTGTCTTCC-3′, reverse: 5′-ACCTGTCTTGGCCGAGGACTAAG-3′; mouse IL-10 forward: 5′-TTCTTTCAAACAAAGGACCAGC-3′, reverse: 5′- GCAACCCAAGTAACCCTTAAAG-3′; mouse iNOS forward: 5′- ACTCAGCCAAGCCCTCACCTAC-3′, reverse: 5′-TCCAATCTCTGCCTATCCGTCTCG-3′; mouse CD86 forward: 5′- ACGGAGTCAATGAAGATTTCCT-3′, reverse: 5′- GATTCGGCTTCTTGTGACATAC; mouse Arg1 forward: 5′- CAGAAGAATGGAAGAGTCAG-3′, reverse: 5′-CAGAAGAATGGAAGAGTCAG-3′; mouse CD206 forward: 5′- CCTATGAAAATTGGGCTTACGG-3′, reverse: 5′-CTGACAAATCCAGTTGTTGAGG-3′. The relative mRNA level was calculated by 2^−ΔΔCt^ method.

### Immunofluorescence

RAW 264.7 cells (1 × 10^5^ per well) were cultured on glass slides in 24 well plate. The cells were washed with PBS and then were fixed with 4% paraformaldehyde. After being washed with PBS again, the cells were treated with 0.1% 100× Triton for 20 min to increase membrane permeability. Afterward, the slices were blocked with goat serum for 1 h and treated with primary antibodies against iNOS (1:500, Abcam) and Arg1 (1:200, Abcam) overnight at 4 °C. Secondary antibodies (Beyotime, Shanghai, China) were incubated for 1 h at room temperature. The slices were counterstained with DAPI and observed with BX53 immunofluorescence microscope (Olympus, Japan).

### Flow cytometry

Mice tracheas were dissected and bronchoalveolar lavage (BAL) fluid was collected by means of slow injection of 1 ml ice-cold PBS into the trachea through a micro syringe, which was repeated three times. The cells from BAL fluid were incubated with CD11b (553311, PE, BD, San Jose, USA) and Ly6G (560,599, APC, BD, San Jose, USA) antibodies for 30 min at 4 °C protected from light to detect the proportion of neutrophils in BAL fluid. Fresh lung tissues were cut into small pieces and digested with collagenase type I (BioFroxx, Einhausen, Germany) and DNase (Solarbio, Beijing, China) solution for 2 h in a water bath at 37 °C. The obtained cell suspensions were filtered through the 200-mesh screen and was centrifuged, re-suspended to prepare the single cell suspension from lung. The cells were co-stained with F4/80 (565,410, PE, BD, San Jose, USA) and CD11b (561,114, PERCP-Cy5.5, BD, San Jose, USA) antibodies for 30 min at 4 °C protected from light to further detect the proportion of macrophages in the lung and to evaluate the efficacy of depletion of macrophage. RAW 264.7 cells were collected, suspended into single cell suspensions, and fixed with Perm/Fix solution (eBoscience, San Diego, USA). Subsequently, the prepared cells were intracellular stained with iNOS (53-5920-80, Alexa Fluro 488, eBoscience, San Diego, USA) or CD206 (17-2061-82, APC, eBoscience, San Diego, USA) antibodies for 30 min at 4 °C protected from light to further measure the proportion of M1 or M2 macrophages. The signals were acquired on a flow cytometer (BD Biosciences, New Jersey, USA). The acquired data were analyzed and visualized using FlowJo v10.4.

### Transfection

RAW 264.7 cells cultured in 6-well plate were transfected with negative control (NC) siRNA or TRAF1 siRNA (Hanbio, Shanghai, China) using HiPerFect Transfection Reagent (Qiagen, Germany) according to manufacture instruction. The sequences of siRNA were listed as follows: TRAF1 siRNA1 forward: 5′-GGAGAAGGUUCACUCUGAUTT-3′, reverse: 5′-AUCAGAGUGAACCUUCUCCTT′; TRAF1 siRNA2 forward: 5′-GGUGGUGGAAUUACAGCAATT-3′, reverse: 5′-UUGCUGUAAUUCCACCACCTT-3′; TRAF1 siRNA3 forward: 5′-CCAGAACAACCGAGAGCAUTT-3′, reverse: 5′-AUGCUCUCGGUUGUUCUGGTT-3′; NC siRNA forward: 5′-UUCUCCGAACGUGUCACGUTT-3′, reverse: 5′-ACGUGACACGUUCGGAGAATT-3′. TRAF1 expression plasmid (PPL, Nanjing, China) transiently transfected into RAW264.7 using X-tremeGENE™ HP DNA Transfection Reagent (Roche, Mannheim, Germany) following manufacturer’s protocol. After 24 h of transfection, cells were treated with 1 μg/ml LPS for 12 h, followed by treatment of exosomes for another 24 h.

### Statistical analysis

The data were displayed with GraphPad Prism 8.0 and as mean ± SEM. Student’s *t* test or One-way analysis of variance (ANOVA), followed by Tukey’s post hoc analysis was used for calculation of statistical differences. *p* values less than 0.05 (*p* < 0.05) were considered to be significant.

## Results

### Characterization of hUCMSC-Exo

**Fig. 1:**
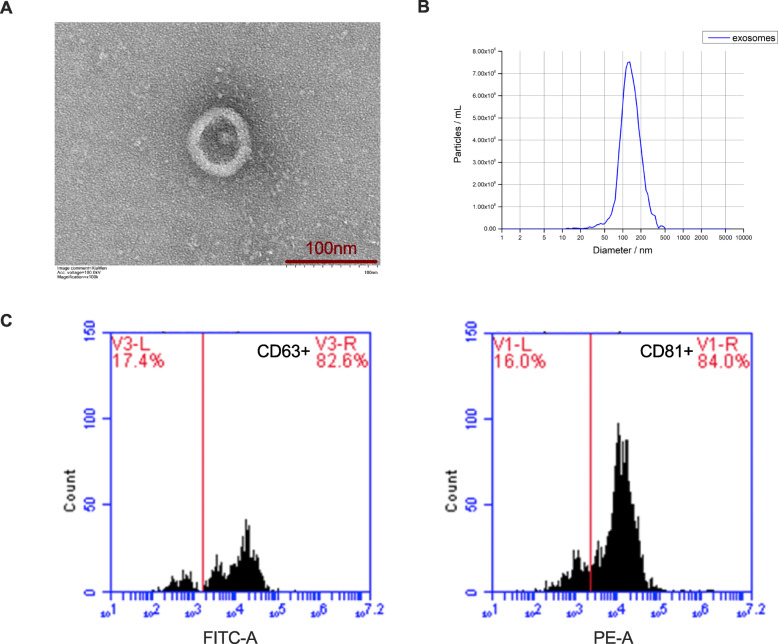
hUCMSC-Exo characterization. **a** Transmission electron microscopy analysis of vesicles derived from hUCMSCs (scale bar = 100 nm). **b** Nanoparticle tracking analysis of vesicles derived from hUCMSCs. **c** Exosome representative markers CD63 and CD81 were detected by flow cytometry analysis

hUCMSC-Exo were extracted and TEM analyses showed the morphology of hUCMSC-Exo displayed a complete structure and a near spherical shape (Fig. 1a). The nanoparticle tracking analysis (NTA) showed that the peak diameter of hUCMSC-Exo was 127.7 nm (Fig. 1b). Flow cytometry analysis confirmed that the exosomes exhibited positive representative markers of CD63 (82.6%) and CD81 (84%) (Fig. 1c). These results indicated that the exosomes from hUCMSC were successfully extracted.

### MSC-Exo inhibited features of OVA/CFA-induced SSRA in mice

**Fig. 2:**
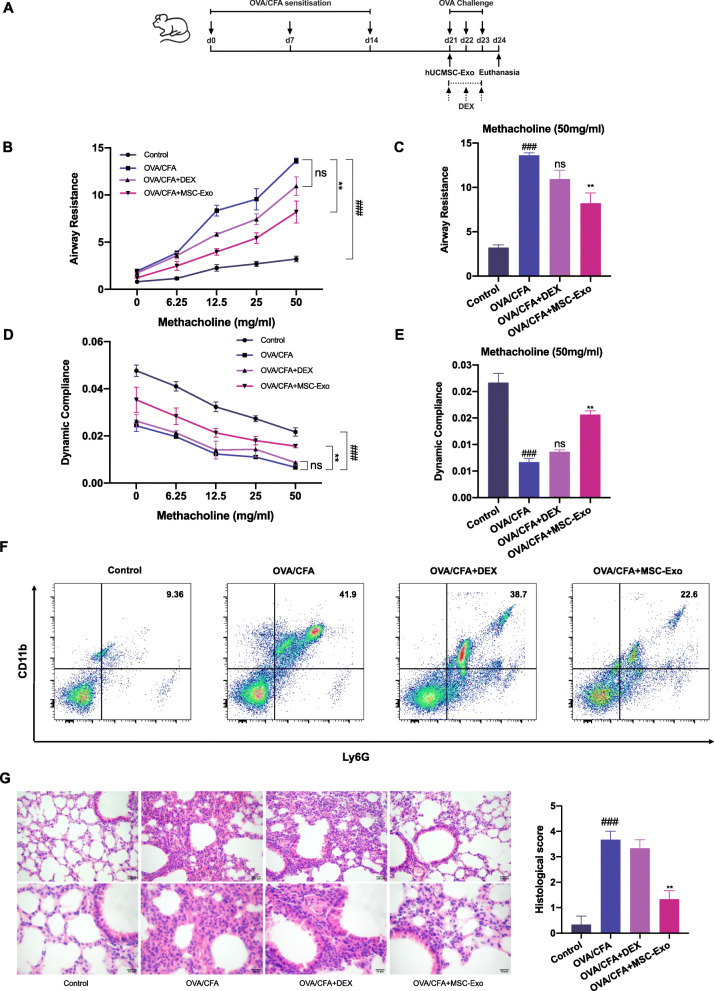
Therapeutic effect of MSC-Exo on OVA/CFA-induced SSRA in mice. **a** The experimental flowchart of construction of mouse model and MSC-Exo treatment. **b** The change of respiratory system resistance in response to different doses of methacholine. **c** The change of respiratory system resistance in response to 50 mg/ml methacholine. **d** The change of lung dynamic compliance in response to different doses of methacholine. **e** The change of lung dynamic compliance in response to 50 mg/ml methacholine. **f** Flow cytometry analysis of neutrophils (CD11b^+^Ly6G^+^) in BAL fluid. **g** H&E staining and histological score evaluation on lung tissues. Above: magnification, × 200; below: magnification, × 400. Data are presented as the mean ± SEM (*n* = 3) (#*p* < 0.05, ##*p* < 0.01, ###*p* < 0.001 vs. the control group; *p < 0.05, **p < 0.01, ***p < 0.001 vs. the OVA/CFA group)

We generated an OVA/CFA-induced SSRA mice and injected exosomes into the bronchus according to the experimental protocol shown as Fig. 2a. We first evaluated the effects of MSC-Exo on lung function. Compared with the control group, pulmonary resistance was more sever and lung dynamic compliance was lower in the OVA/CFA group, and DEX failed to reverse AHR. Interestingly, MSC-Exo treatment reduced pulmonary resistance and reversed lung dynamic compliance (Fig. 2b–e). This result indicated that MSC-Exo treatment ameliorated AHR in OVA/CFA-induced SSRA. Neutrophil infiltration in BAL fluid was elevated in severe asthma patients or murine model [2, 28]. We then assessed neutrophils (CD11b^+^Ly6G^+^) infiltration in BAL fluid. We found that neutrophil numbers were not reduced by DEX treatment in the OVA/CFA group (OVA/CFA+DEX) but was decreased in the MSC-Exo treated group (OVA/CFA+MSC-Exo) compared with OVA/CFA group (Fig. 2f). Furthermore, H&E staining on lung tissues also showed increased neutrophil infiltration in the airways, lung parenchyma, and blood vessels in the OVA/CFA group, with a higher histological score. In the OVA/CFA+MSC-Exo group, neutrophil infiltration was markedly reduced, with reduced histological score (Fig. 2g). These results indicated that MSC-Exo was effective in reducing inflammation in SSRA in vivo.

### Macrophage depletion reduced the effect of MSC-Exo on OVA/CFA-induced SSRA in mice

**Fig. 3:**
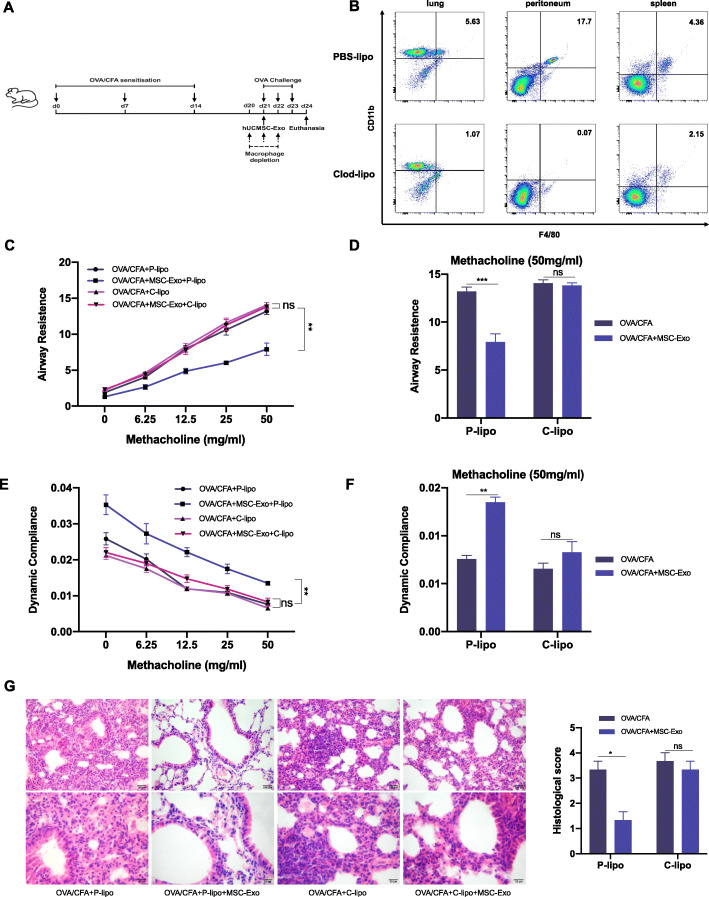
The therapeutic effect of MSC-Exo on OVA/CFA-induced SSRA asthmatic mice is macrophage-dependent. **a** The experimental schematic of macrophage depletion experiment. **b** Flow cytometry analysis of macrophages (F4/80^+^CD11b^+^) in the lung, peritoneal fluid, and spleen from different groups. **c** The change of respiratory system resistance in response to increasing concentrations of methacholine. **d** The change of respiratory system resistance in response to 50 mg/ml methacholine. **e** The change of lung dynamic compliance in response to increasing concentrations of methacholine. **f** The change of lung dynamic compliance in response to 50 mg/ml methacholine. **g** H&E staining and histological score evaluation on lung tissues. Data are presented as the mean ± SEM (*n* = 3) (*p < 0.05, **p < 0.01, ***p < 0.001 vs. the OVA/CFA+P-lipo group)

Since macrophages play an important role in respiratory diseases, we next depleted macrophages in mice through intraperitoneal injection of clodronate liposomes. The macrophage depletion experiment was conducted according to the experimental protocol shown as Fig. 3a. Administration of clodronate liposomes effectively depleted macrophages in the lung, peritoneum, and spleen (Fig. 3b). In the PBS liposome-treated group, MSC-Exo reduced AHR and inflammatory cell infiltration, consistent with the results above. However, this reduction in inflammation by MSC-Exo in OVA/CFA-induced SSRA mice was absent in macrophage-depleted group, with minimal improvement in airway resistance and dynamic compliance (Fig. 3c–f). Moreover, the beneficial effect of MSC-Exo on histopathology was also reduced in macrophage-depleted group (Fig. 3g). In general, these results demonstrated that macrophages were necessary for the therapeutic effect of MSC-Exo on OVA/CFA-induced SSRA in mice.

### MSC-Exo ameliorated inflammation and regulated macrophage polarization in OVA/CFA-induced SSRA in mice

**Fig. 4:**
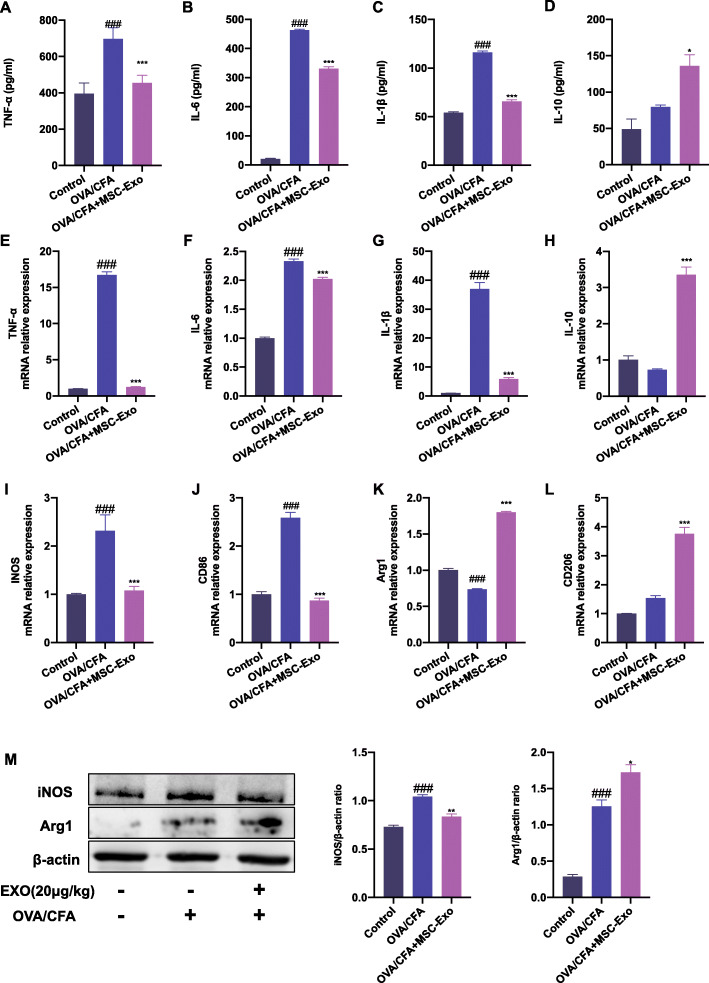
Effect of MSC-Exo on inflammation and macrophage polarization in OVA/CFA-induced SSRA in mice. **a**–**d** The result of ELISA about protein levels of pro-inflammatory cytokines TNF-α, IL-6, IL-1β, and anti-inflammatory cytokines IL-10 of supernatant from lung tissues. **e**–**h** qRT-PCR results about mRNA level of cytokines TNF-α, IL-6, IL-1β, and IL-10 from lung tissues. **i**–**l** qRT-PCR results about mRNA level of M1 markers iNOS, CD86, and M2 marker Arg1 and CD206 from lung tissues. **m** Western blot and quantification results of iNOS and Arg1 in lung. β-actin was used as internal reference. Data are presented as the mean ± SEM (*n* = 3) (^#^*p* < 0.05, ^##^*p* < 0.01, ^###^*p* < 0.001 vs. the control group; *p < 0.05, **p < 0.01, ***p < 0.001 vs. the OVA/CFA group)

Macrophage-mediated inflammatory response and the polarization state of macrophage play a central role in the development and severity of asthma [4]. To further evaluate the regulatory effect of MSC-Exo on macrophages in OVA/CFA-induced SSRA in mice, we assessed the production of inflammatory cytokines in the supernatant from lung tissues. The levels of pro-inflammatory and M1 cytokines signature TNF-α, IL-6, and IL-1β were significantly increased in the OVA/CFA group. MSC-Exo significantly decreased the levels of these cytokines compared with OVA/CFA group (Fig. 4a–c, e–g). In contrast, compared with the OVA/CFA group, the level of anti-inflammatory cytokine and M2 marker IL-10 was increased in the MSC-Exo group (Fig. 4d, h). To further understand how MSC-Exo modulates macrophage polarization in OVA/CFA-induced asthmatic mice, we assessed the expressions of M1 markers iNOS, CD86, and M2 markers Arg1 and CD206 in lung tissue. We found that the mRNA levels of iNOS and CD86 were significantly decreased in the MSC-Exo group compared with the OVA/CFA group. The mRNA levels of Arg1 and CD206 were increased by the treatment of MSC-Exo (Fig. 4i–l). Consistently, the protein level of iNOS was downregulated while the level of Arg1 protein expression was upregulated in the MSC-Exo group compared with the OVA/CFA group (Fig. 4m). These results indicated that administration of MSC-Exo inhibited inflammation and reshaped the macrophage polarization in lung of mice with OVA/CFA-induced SSRA.

### MSC-Exo exhibited anti-inflammatory and polarization regulatory effects on LPS-stimulated RAW 264.7 cells

**Fig. 5:**
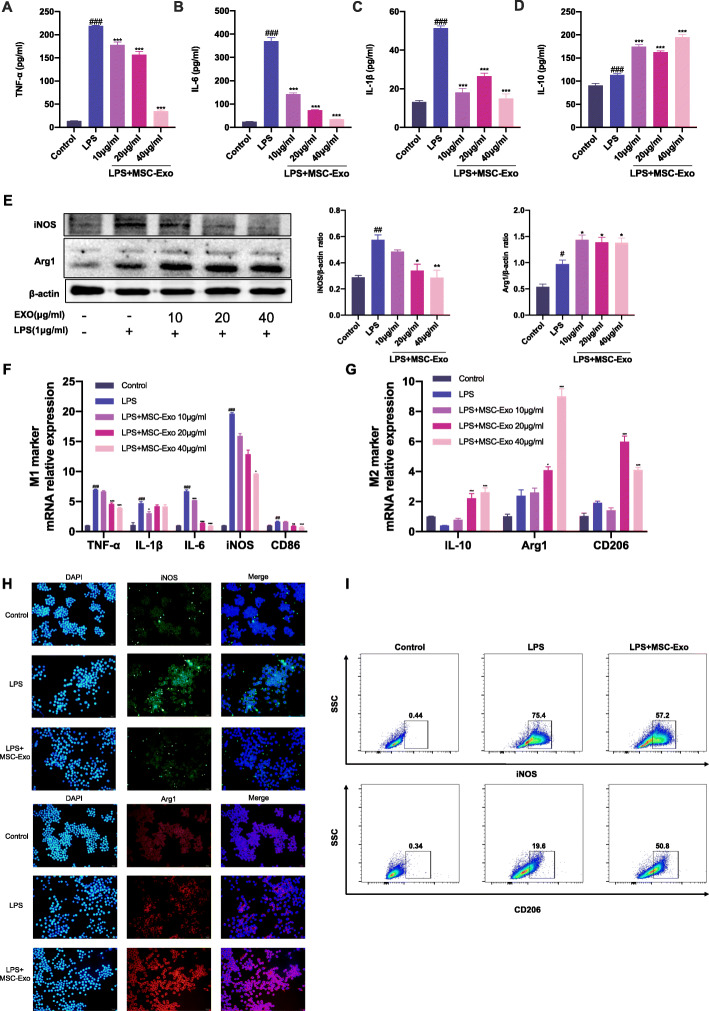
Effect of MSC-Exo on inflammation and macrophage polarization in LPS-stimulated RAW 264.7 cells. **a**–**g** RAW 264.7 cells were stimulated by LPS for 12 h, followed by the treatment of MSC-Exo (10, 20, 40 μg/ml) for 24 h. **a**–**d** Levels of TNF-α, IL-6, IL-1β, and IL-10 in culture supernatants of RAW 264.7 cells of different groups. **e** Western blotting image and quantification of iNOS and Arg1 expression in RAW 264.7 cells of different groups. β-actin was used as internal reference. **f**, **g** qRT-PCR analysis of mRNA level of M1 markers TNF-α, IL-6, IL-1β, iNOS, CD86, and M2 markers IL-10, Arg1, and CD206 in RAW 264.6 cells of different groups. **h**, **i** RAW 264.7 cells were stimulated by LPS for 12 h, followed by the treatment of MSC-Exo (20 μg/ml) for 24 h. **h** Representative images of immunofluorescence staining of iNOS and Arg1 in RAW 264.7 cells of different groups. **i** Representative images of flow cytometry analysis of iNOS and CD206 in RAW 264.7 cells of different groups. Data are presented as the mean ± SEM (*n* = 3) (^#^*p* < 0.05, ^##^*p* < 0.01, ^###^*p* < 0.001 vs. the control group; *p < 0.05, **p < 0.01, ***p < 0.001 vs. the LPS group)

We have found that MSC-Exo treatment ameliorated OVA/CFA-induced SSRA in mice and macrophages were an indispensable participant in this progress. In OVA/CFA-induced SSRA mice, MSC-Exo promoted M2 macrophage polarization in lung. To determine if MSC-Exo directly impact macrophage phenotypes and cytokine productions, we used a macrophage polarization model. RAW 264.7 cells were treated with LPS for 12 h followed by MSC-Exo treatment for 24 h. While LPS induced significant production of TNF-α, IL-6, IL-1β, and iNOS, MSC-Exo significantly reduced the levels of these pro-inflammatory mediators and increased the expression of IL-10 and Arg1 (Fig. 5a–e). The mRNA levels of M1 markers TNF-α, IL-6, IL-1β, iNOS, CD86 were downregulated and the mRNA levels of M2 markers IL-10, Arg1, and CD206 were upregulated in MSC-Exo-treated and LPS-stimulated RAW264.7 cells (Fig. 5f, g). Immunofluorescent staining also showed decreased M1 marker iNOS staining, while the M2 marker Arg1 staining was more prominent in MSC-Exo group compared with LPS group (Fig. 5h). Furthermore, the flow cytometric analyses showed that the proportion of iNOS^+^ M1 macrophages was decreased in MSC-Exo group compared with LPS group. The proportion of CD206^+^ M2 macrophages was increased in MSC-Exo group compared with LPS group (Fig. 5i). These results demonstrated that MSC-Exo inhibited M1 macrophage polarization and promoted M2 macrophage polarization in LPS-stimulated RAW 264.7 cells.

### TRAF1 was involved in MSC-Exo mediated macrophage polarization

**Fig. 6:**
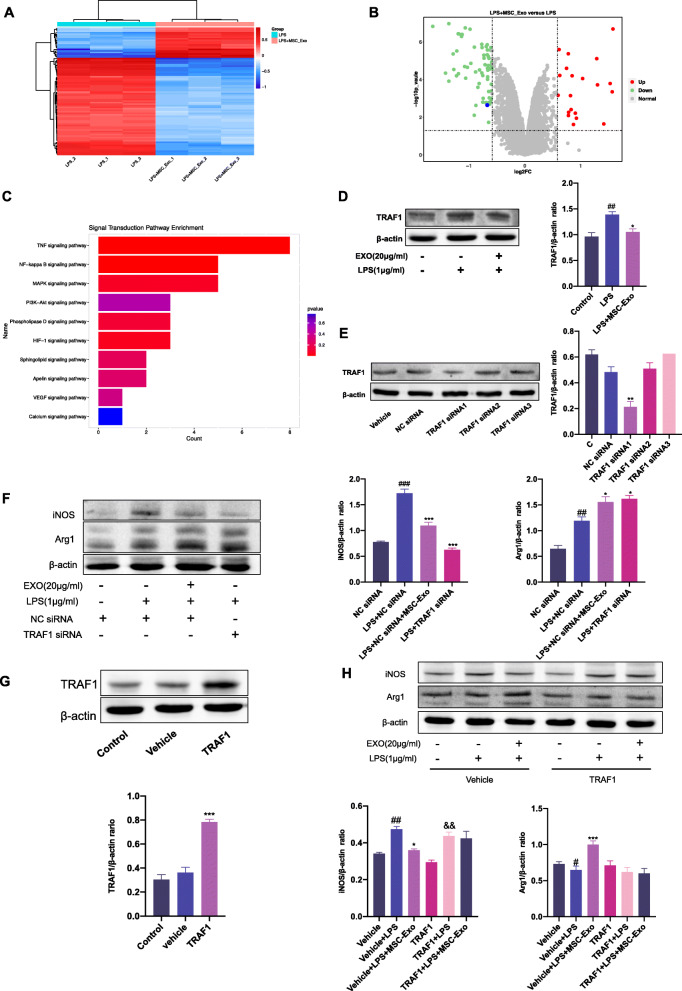
Effect of MSC-Exo on macrophage polarization is related to TRAF1. RAW 264.7 cells were stimulated by LPS for 12 h, followed by the treatment of MSC-Exo (20 μg/ml) for 24 h. Samples from LPS group and MSC-Exo group were collected for proteomics. **a** Heat map of significantly differential expression proteins in LPS+MSC-Exo group compared with LPS group. **b** Volcano plot of significantly differential expression proteins in LPS+MSC-Exo group compared with LPS group (vertical dotted lines, fold change > 1.5-fold; horizontal dotted line, *p* < 0.05; blue point, TRAF1). (**c**) Top 10 of signaling transduction of KEGG pathway enrichment analysis. **d** The expression level of TRAF1 was detected by Western blotting and quantified by ImagJ. β-actin was used as internal reference (^#^*p* < 0.05, ^##^*p* < 0.01, ^###^*p* < 0.001 vs. the control group; *p < 0.05, **p < 0.01, ***p < 0.001 vs. the LPS group). (**e**) RAW 264.7 cells were transfected with TRAF1 siRNA or NC siRNA. The knockdown effect of siRNA on TRAF1 was detected by Western blotting. β-actin was used as internal reference (*p < 0.05, **p < 0.01, ***p < 0.001 vs. the NC siRNA group). (**f**) The expression levels of iNOS and Arg1 were detected using Western blotting and quantified using ImagJ. β-actin was used as internal reference (^#^*p* < 0.05, ^##^*p* < 0.01, ^###^*p* < 0.001 vs. the NC siRNA group; *p < 0.05, **p < 0.01, ***p < 0.001 vs. the LPS+NC siRNA group.) Data are presented as the mean ± SEM (*n* = 3). **g** TRAF1 overexpression transfection of RAW 264.7 cells. The expression level of TRAF1 was detected using Western blotting and quantified by ImagJ. β-actin was used as internal reference (*p < 0.05, **p < 0.01, ***p < 0.001 vs. the vehicle group). **h** After TRAF1 overexpression transfection, macrophages were stimulated with LPS for 12 h, followed by treatment with MSC-Exo for 24 h. The protein levels of iNOS and Arg1 were measured by Western blotting and quantified by ImageJ software. Data are presented as the mean ± SEM (*n* = 3) (^#^*p* < 0.05, ^##^*p* < 0.01, ^###^*p* < 0.001 vs. the vehicle group; *p < 0.05, **p < 0.01, ***p < 0.001 vs. the vehicle+LPS group; ^&^*p* < 0.05, ^&&^*p* < 0.01, ^&&&^*p* < 0.001 vs. the TRAF1 group)

Our results indicated that MSC-Exo regulated macrophage polarization both in SSRA murine model and macrophage polarization model. To better understand the changes in protein expression profile of MSC-Exo treated LPS-stimulated macrophages, we performed TMT-labeled quantitative proteomics using samples from LPS group and LPS+MSC-Exo group. We identified 6761 quantified protein groups, among which 84 proteins were significantly regulated. Cluster analysis showed differentially expression proteins, and the heatmap demonstrated two distinct groups (LPS group and LPS+MSC-Exo group) (fold change > 1.5 or < 0.67, *p* < 0.05) (Fig. 6a). The volcano plot exhibited that among the 84 differential proteins, 21 proteins were upregulated and 63 proteins were downregulated in LPS+MSC-Exo group compared with LPS group (Fold change > 1.5 or < 0.67, *p* < 0.05) (Fig. 6b). In the top 10 of Kyoto Encyclopedia of Genes and Genomes (KEGG) pathway analysis on signal transduction level (Fig. 6c), multiple pathways, including the TNF, NF-κB, and PI3K-AKT signaling pathways, are well characterized in macrophage polarization. By mining the proteins annotated to the enrichment pathways, we found that TRAF1 was annotated to the macrophage polarization-related pathways multiple times and had significant difference. Western blotting confirmed the expression of TRAF1 was upregulated in LPS group and was downregulated after MSC-Exo treatment, consistent with the result of change of expression level of TRAF1 in proteomics (Fig. 6d). To assess the role of TRAF1 in MSC-Exo-mediated inhibition of M1 polarization, we artificially silenced TRAF1 expression using specific siRNAs. TRAF1-siRNA1 showed the highest knockdown efficacy and was used for the subsequent experiments (Fig. 6e). Knockdown of TRAF1 reduced the protein expression of iNOS and promote the expression of Arg1, which was consistent with MSC-Exo (Fig. 6f). In addition, overexpression of TRAF1 experiment was conducted. The efficiency of TRAF1 overexpression was shown in Fig. 6g. Overexpression of TRAF1 reduced the effect of MSC-Exo on iNOS and Arg1 (Fig. 6h). These results indicated that the polarization regulatory effect of MSC-Exo on LPS-stimulated RAW 264.7 cells was TRAF1 dependent.

### MSC-Exo regulated activation of NF-κB and PI3K/AKT signaling pathways which was mediated by inhibition of TRAF1

**Fig. 7:**
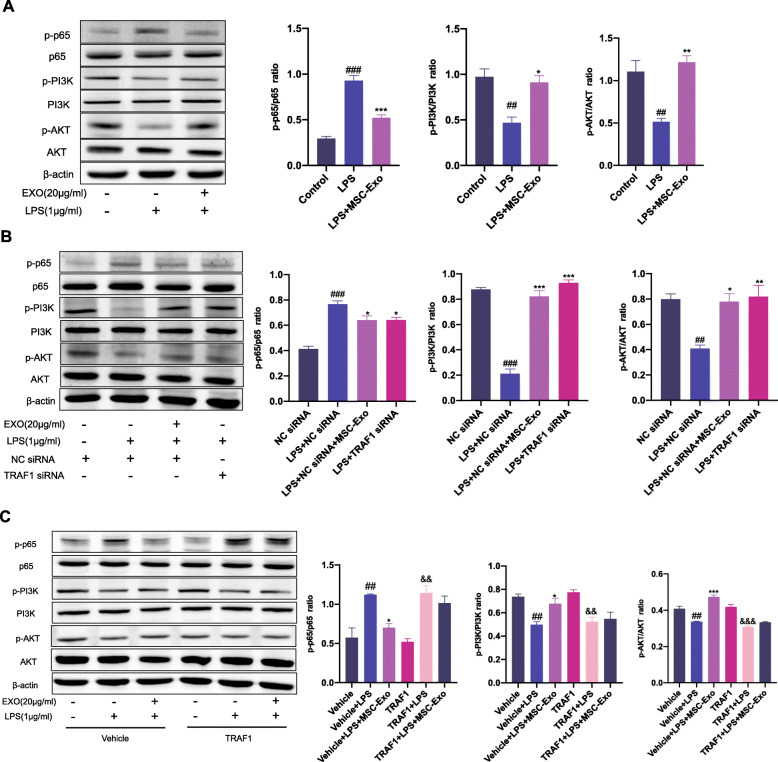
Effect of MSC-Exo on NF-κB and PI3K/AKT signaling pathways was mediated by inhibition of TRAF1. **a** RAW 264.7 cells were treated with LPS for 12 h, followed by treatment with MSC-Exo for 24 h. The protein levels of p-p65, p65, p-PI3K, PI3K, p-AKT, and AKT were measured by Western blotting and quantified by ImageJ software. **b** After transfection with siRNA, macrophages were stimulated with LPS for 12 h, followed by treatment with MSC-Exo for 24 h. The protein levels of p-p65, p65, p-PI3K, PI3K, p-AKT, and AKT were measured by Western blotting and quantified by ImageJ software. Data are presented as the mean ± SEM (*n* = 3) (^#^*p* < 0.05, ^##^*p* < 0.01, ^###^*p* < 0.001 vs. the NC siRNA group; *p < 0.05, **p < 0.01, ***p < 0.001 vs. the LPS+NC siRNA group). **c** After TRAF1 overexpression transfection, macrophages were stimulated with LPS for 12 h, followed by treatment with MSC-Exo for 24 h. The protein levels of p-p65, p65, p-PI3K, PI3K, p-AKT, and AKT were measured by Western blotting and quantified by ImageJ software. Data are presented as the mean ± SEM (*n* = 3) (^#^*p* < 0.05, ^##^*p* < 0.01, ^###^*p* < 0.001 vs. the vehicle group; *p < 0.05, **p < 0.01, ***p < 0.001 vs. the vehicle+LPS group; ^&^*p* < 0.05, ^&&^*p* < 0.01, ^&&&^*p* < 0.001 vs. the TRAF1 group)

We have identified the key role of TRAF1 in the regulation effect of MSC-Exo on macrophage polarization. To further investigate the mechanism, we analyzed and mined the results of proteomics. According to the KEGG pathway enrichment analysis on signaling transduction level and consideration of the relationship between related pathways and macrophage polarization, NF-κB and PI3K/AKT signaling pathways were explored in the further experiments. MSC-Exo in LPS-stimulated RAW 264.7 cells significantly decreased the phosphorylation of p65 but increased the phosphorylation of PI3K and AKT. This indicated that MSC-Exo inhibited the activation of NF-κB signaling pathway and promoted the activation of PI3K/AKT pathway (Fig. 7a). Since we have found that TRAF1 plays an important role in macrophage polarization, we used TRAF1 siRNA to further explore the effect of TRAF1 knockdown on the activation of NF-κB and PI3K/AKT signaling pathways. Knockdown of TRAF1 in LPS-stimulated macrophages suppressed the activation of NF-κB signaling pathway and promoted the activation of PI3K/AKT pathway, exhibiting the similar effect of MSC-Exo (Fig. 7b). However, overexpression of TRAF1 significantly diminished the effect of MSC-Exo on NF-κB and PI3K/AKT pathways (Fig. 7c). Taken together, these results indicated that the effect of MSC-Exo treatment on NF-κB and PI3K/AKT signaling pathways was TRAF1 dependent.

### MSC-Exo inhibited TRAF1 in OVA/CFA-induced SSRA in mice

**Fig. 8:**
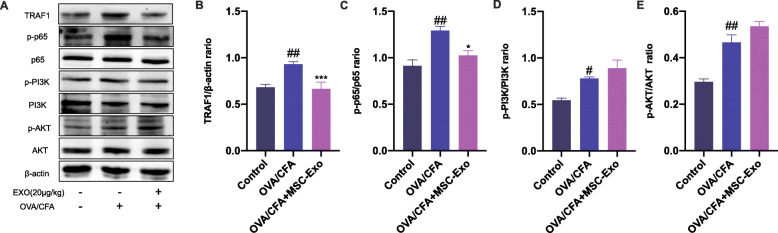
Effect of MSC-Exo on TRAF1, NF-κB, and PI3K/AKT signaling pathways in OVA/CFA-induced SSRA in mice. **a** The protein levels of TRAF1, p-p65, p65, p-PI3K, PI3K, p-AKT, and AKT in lung tissue of different groups were measured by Western blot. **b**–**e** All the bands were quantified by ImageJ software. The ratio of TRAF1/β-actin, p-p65/p65, p-PI3K/PI3K, and p-AKT/AKT were displayed. Data are presented as the mean ± SEM (*n* = 3) (^#^*p* < 0.05, ^##^*p* < 0.01, ^###^*p* < 0.001 vs. the control group; *p < 0.05, **p < 0.01, ***p < 0.001 vs. the OVA/CFA group)

We have found that MSC-Exo treatment inhibited M1 macrophage polarization and promoted M2 macrophage polarization in OVA/CFA-induced SSRA in mice and explored the underlying mechanism which MSC-Exo regulated activation of NF-κB and PI3K/AKT signaling pathways through inhibiting TRAF1 in in vitro model. Then, we evaluated the protein expression changes of TRAF1 and the related pathways in OVA/CFA-induced SSRA in mice. We found that the expression of TRAF1 was increased in lung tissue from OVA/CFA group compared with control group. Consistent to in vitro study, MSC-Exo treatment inhibited the expression of TRAF1 in OVA/CFA-induced asthmatic mice. We also showed that MSC-Exo treatment significantly decreased the phosphorylation of p65 compared with OVA/CFA group. However, MSC-Exo treatment only slightly increased the activation of PI3K/AKT signaling pathways compared with OVA/CFA group (Fig. 8a–e).

## Discussion

In this study, we established a murine model of OVA/CFA-induced SSRA. We found that the experimental model showed neutrophil infiltration and airway hyper-responsiveness, with no response to dexamethasone. All these characteristics are consistent with the clinical manifestations of patients with SSRA [29]. We propose a new strategy for the treatment of SSRA, using exosomes to shift from MSC-based cell transplantation to cell-free therapy. We demonstrated for the first time the therapeutic effect of MSC-Exo in SSRA. However, the effect of MSC-Exo treatment was reversed in the macrophage exhaustion model, which revealed that macrophages are the main downstream target of MSC-Exo. Our work further confirmed that MSC-Exo effectively regulate the polarization of macrophages and reduce inflammatory cascades and the underlying mechanism may be regulating activation of NF-κB and PI3K/AKT signaling pathways via targeting TRAF1.

In SSRA, the persistence and excessive inflammatory response are associated with decreased control and increased exacerbations. The infiltration of inflammatory cells and the accumulation of pro-inflammatory cytokines cause an inflammatory cascade in the lungs, which aggravates the airway inflammation, lung tissue damage, and airway remodeling [30]. Vigorous research has been focused on the mechanisms underpinning this heightened inflammation and potential therapeutics. Accumulating studies have demonstrated the therapeutic potentials of MSCs for many immune-related diseases. MSCs have been shown to interact with all innate immune cells and promote anti-inflammatory response [31]. In animal models and pre-clinical experimental studies, MSC transplantation has a beneficial therapeutic effect on pulmonary inflammatory diseases [13, 14, 32]. However, there are still many drawbacks and risks in MSC transplant therapies [33]. Increasing evidence have highlighted that the immunotherapy effects of MSCs are driven by the paracrine signal transduction between cells [34]. Exosomes are nano-sized extracellular vesicles derived from maternal cells. Through intracellular transfer of bioactive cargo such as proteins, enzymes, nucleic acids, and metabolites, exosomes exert direct effects on these paracrine pathways [35]. However, the specific mechanism of MSC-Exo in immune regulation has not been elucidated in detail. In this study, we investigated the effects of exosomes derived from MSCs in reducing inflammation. Exosomes have many ideal advantages, including no immune rejection, no tumorigenicity, and no vascular obstruction [36]. Our data firmly demonstrated that MSC-Exo improved lung inflammation and airway hyper-responsiveness in OVA/CFA-induced murine model of SSRA.

Macrophages are the central mediator of lung inflammation [37], and macrophage polarization has a profound impact on the pathogenesis of asthma. Macrophages can be polarized into either M1 or M2 phenotypes and are involved in the initiation and regression of inflammation, which is closely related to the development of asthma [38]. To further assess the role of macrophages in MSC-Exo treatment, we applied the macrophage depletion model. Of note, we found that the therapeutic effect of MSC-Exo largely depends on the interaction with macrophages. The process of macrophage polarization involves complex interactions between various cytokines, chemokines, transcription factors, and immune regulatory cells [39]. Pro-inflammatory factors secreted by M1 macrophages can aggravate airway hyper-responsiveness, play a positive regulatory role in the chemotactic recruitment of neutrophils, and at the same time promote neutrophils to release inflammatory factors and amplify inflammatory effects, as well as damage lung tissue. Several studies have suggested that M1 cytokines increases significantly in patients with severe asthma [40]. Besides, M1 markers are also highly expressed in the alveolar lavage fluid of patients with steroid-resistant asthma [9]. The above results indicated that M1 macrophages play a crucial role in the occurrence and development of SSRA. However, unlike M1 macrophages, M2 macrophages usually exert anti-inflammatory functions. The cytokine IL-10 secreted by M2 macrophages can effectively reduce airway inflammation, promote lung injury repair, and attenuate airway remodeling after asthma [4]. Therefore, the reversal of M1 to M2 phenotype has been shown to be a potential treatment strategy of SSRA. In this study, we confirmed that MSC-Exo can effectively convert macrophages from M1 to M2 phenotype in the asthmatic niche, whether in SSRA murine model and LPS-stimulated cell model.

To further investigate the potential mechanism of MSC-Exo in inhibiting inflammation by regulating macrophage polarization, we performed TMT-labeled quantitative proteomics using macrophage polarization model. Tumor necrosis factor receptor-associated factor 1 (TRAF1) was found to be significantly downregulated in LPS-stimulated macrophages with MSC-Exo treatment. TRAF1 is an important cytoplasmic adaptor protein involved in regulating immunity, inflammation, and apoptosis [41]. A study by Wan et al. showed that TRAF1 ablation reduced inflammation and attenuated lung damage in LPS-induced acute lung injury [42]. TRAF1 is a critical signaling factor in NF-κB activation in B cells and T cells [43]. Activation of NF-κB promotes macrophage polarization to M1 [44]. Consistently, our results showed that the knockdown of TRAF1 decreased activation of NF-κB and inhibited M1 polarization in LPS-stimulated macrophages, similar to the effect of MSC-Exo. Moreover, MSC-Exo also inhibited expression of TRAF1 and activation of NF-κB signaling pathway in our SSRA model. Activation of PI3K/AKT signaling pathway is important for M2 polarization [45–48]. We also showed that the PI3K/AKT signaling pathway modulated macrophage polarization, as TRAF1 knockdown increased PI3K/AKT activation and promoted M2 polarization in LPS-stimulated macrophages, similar to that observed with MSC-Exo. However, the effect of MSC-Exo on PI3K/AKT activation was slight in OVA/CFA-induced asthmatic mice compared with OVA/CFA group. This inconsistency may be caused by the fact that macrophages are the main target cells of exosomes. In LPS-stimulated macrophages, overexpression of TRAF1 abrogated the effects of MSC-Exo on polarization, NF-κB, and PI3K/AKT signaling pathways. Taken together, our data indicated that TRAF1 was important in MSC-Exo-mediated effect on macrophage polarization via NF-κB and PI3K/AKT signaling pathways.

In this study, the therapeutic effect of MSC-Exo in SSRA was explored for the first time. We focused on the macrophage polarization remodeling effect of MSC-Exo and found that TRAF1 was the key actor in this process. This study provides a valuable contribution in exploring mechanism involved in the macrophage remodeling function of MSC-Exo in SSRA.

## Conclusions

We demonstrated that MSC-Exo ameliorated OVA/CFA-induced SSRA through macrophage polarization remodeling. MSC-Exo treatment modulated NF-κB and PI3K/AKT signaling pathways by inhibiting TRAF1 expression and promoted macrophage M2 polarization. This study provides a novel strategy for treatment of SSRA.

## Data Availability

The datasets used and analyzed during our study are available from the corresponding author on reasonable request.

## Abbreviations

SSRA: Severe, steroid-resistant asthma
MSC: Mesenchymal stem cell
hUCMSC: Human umbilical cord mesenchymal stem cell
MSC-Exo: Exosomes derived from mesenchymal stem cell
AHR: Airway hyper responsiveness
TMT: Tandem mass tags
TRAF1: Tumor necrosis factor receptor-associated factor 1
LPS: Lipopolysaccharide
TNF-α: Tumor necrosis factor alpha
IL-6: Interleukin-6
iNOS: Inducible nitric oxide synthase
Arg1: Arginase-1
OVA: Ovalbumin
CFA: Complete Freud’s adjuvant
DEX: Dexamethasone
C-lipo: Clodronate liposomes
H&E: Hematoxylin and eosin
BAL: Bronchial alveolar lavage
TEM: Transmission electron microscopy
NTA: Nanoparticle tracking analysis
KEGG: Kyoto Encyclopedia of Genes and Genomes
